# Near-infrared-spectroscopic study on processing of sounds in the brain; a comparison between native and non-native speakers of Japanese

**DOI:** 10.3109/00016489.2016.1139745

**Published:** 2016-02-15

**Authors:** Koichi Tsunoda, Sotaro Sekimoto, Kenji Itoh

**Affiliations:** ^a^ Department of Artificial Organs and Medical Creations, National Hospital Organization, National Institute of Sensory Organs, National Tokyo Medical Center, Tokyo, Japan

**Keywords:** Brain dominance, speech and hearing, Japanese language, mother tongue, Tsunoda’s Theory, Japanese brain, near-infrared spectroscopy

## Abstract

**Conclusions** The result suggested that mother tongue Japanese and non- mother tongue Japanese differ in their pattern of brain dominance when listening to sounds from the natural world—in particular, insect sounds. These results reveal significant support for previous findings from Tsunoda (in 1970).

**Objectives** This study concentrates on listeners who show clear evidence of a ‘speech’ brain vs a ‘music’ brain and determines which side is most active in the processing of insect sounds, using with near-infrared spectroscopy.

**Methods** The present study uses 2-channel Near Infrared Spectroscopy (NIRS) to provide a more direct measure of left- and right-brain activity while participants listen to each of three types of sounds: Japanese speech, Western violin music, or insect sounds. Data were obtained from 33 participants who showed laterality on opposite sides for Japanese speech and Western music.

**Results** Results showed that a majority (80%) of the MJ participants exhibited dominance for insect sounds on the side that was dominant for language, while a majority (62%) of the non-MJ participants exhibited dominance for insect sounds on the side that was dominant for music.

## Introduction

It is generally accepted that there are lateral asymmetries in the activities of the cerebral hemispheres during the perception of different types of sound. The left is generally more active during speech perception, while the right is more active during the perception of many other types of sound, including tonal musical sounds [1,2]. In the early 1970s, a Japanese physician, Tadanobu Tsunoda, obtained experimental results that indicated a distinct difference between native speakers of Japanese and speakers of other Asian and Western languages in how the right and left cerebral hemispheres process vowels and sounds from nature, such as those made by insects [3,4]. In particular, these sounds tended to be processed in the left brain for Japanese speakers and in the right brain for speakers of the other languages. For vowels, the difference was attributed to the peculiarities of Japanese, in which vowel sounds form meaningful monosyllabic words and are processed in the dominant hemisphere, similar to consonants and complete syllables in other languages [5–9]. The characteristic patterns were thought to develop in native speakers of Japanese due to these specific linguistic characteristics, with no genetic factor involved. The lateralization of sounds from nature was also thought to be related to cultural factors, with native Japanese speakers processing these sounds primarily in the ‘language brain’, while native speakers of the other language processed them primarily in the ‘music’ or ‘non-language brain’.

Tsunoda’s ideas were controversial and have not been widely accepted [10–12]. One possible reason for this is that the methods employed in Tsunoda’s studies were necessarily indirect, involving a key-tapping paradigm and delayed auditory feedback while subjects listened to repeated presentations of brief sounds with durations of 0.1–0.3 s. The goal of the present study is to further examine one of Tsunoda’s findings, using a more direct modern brain-monitoring technique. In particular, we use near-infrared spectroscopy (NIRS) [13] to examine hemispheric dominance in the processing of insect sound in native and non-native Japanese speakers. While the processing of short vowel sounds and its possible relationship to the structures of Japanese and other languages is, of course, also of interest, this is not so clearly amenable to brain-monitoring methods, because the stimuli of interest are necessarily short. Rather we will concentrate on hemispheric dominance in the processing of sounds from nature. The hypothesis to be tested is that insect sounds are processed in the ‘speech’ brain for native Japanese speakers, but in the ‘music’ brain for native speakers of many other languages. In particular, we further examine the processing of insect sounds and its relationship to processing of speech and music. To do this, we compare the change in activity of both the left and right cerebral hemispheres in response to three types of auditory stimuli (speech, music, and insect sounds) between native Japanese speakers (mother-tongue Japanese, MJ) and non-native Japanese speakers (non-Japanese-mother-tongue speakers, non-MJ) through use of NIRS. We concentrate on listeners who show clear evidence of a ‘speech’ brain vs a ‘music’ brain and determine which side is most active in the processing of insect sounds.

## Methods

### Ethics

This research protocol (R10-036R) was approved by the National Hospital Organization, Tokyo Medical Center Ethics Committee (the institutional review board of Tokyo Medical Center). Before participating in the studies, all volunteers provided written informed consent.

### Volunteers

Initially there were 70 volunteers: 44 MJ Japanese and 26 non-MJ Caucasians. From these, any who had previously received facial or brain surgery were excluded from the study. Using this and other criteria, described below, 37 volunteers were excluded from the study. The results reported here are from 33 volunteers (nine Japanese males, mean age = 26.9 years and age range = 23–32; 11 Japanese females, mean age = 28.4 years old and age range = 18–38; five Caucasian males, mean age = 24.2 and range = 21–32; eight Caucasian females, mean age = 24.2 and range = 20–39. Between the Japanese and Caucasian groups there were no group differences in terms of age (*p* = 0.97) or gender (*p* = 0.49). All showed different hemispheric dominance of cerebral blood flow in response to Japanese-language and Western-music stimuli. The MJ volunteers were all Japanese citizens and the Caucasian (non-MJ) volunteers were all non-Japanese citizens, all fluent in their mother tongue (three Russian, two Italian, and one each of Arabic, French, German, Hindi, Persian, Polish, Slovenian, and Swedish) and able to understand Japanese speech.

### Equipment

Measurements were made with a 2-channel NIRS device (HOT-121 NIRS, Hitachi, Tokyo) that was developed to examine oxy-hemoglobin levels of the brain, specifically the frontal lobe, via the forehead, since this area is void of obstruction (hair). This device includes velocity sensors that detect head motions. The NIRS system can determine brain oxy-hemoglobin values of the superficial layers (3 cm diameter and ca. 2 cm deep) in each channel. We used two channels (left and right). The probes were attached to the volunteer’s forehead just below the hairline. The two channels were, thus, placed to record from and compare activity in left and right frontal lobes from their blood oxy-hemoglobin levels. By using NIRS, we eliminated the acoustic background noise and discomfort associated with fMRI and were able to perform listening tests in a relatively natural (sitting) setting.

**Figure 1.**
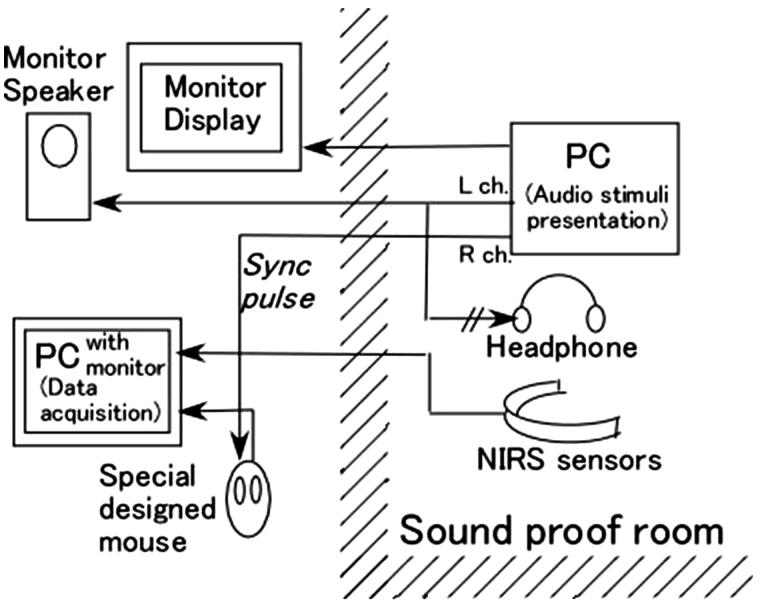
Schematic of the experimental set-up. See text for description.

The experimental set-up is illustrated in Figure 1. Two computers were used to perform event-related NIRS recordings. The first was used for NIRS signal recording using the measurement software of the HOT-121 system (Hitachi Co. Ltd., Tokyo, Japan). The second computer was used for stimulus presentation via Microsoft PowerPoint. A pulse synchronized with the start of each stimulus was used to synchronize the data recording with the stimulus presentation. This was accomplished using a specially designed device that simulated a mouse**-**button press in response to the synchronization pulse. Stimuli were presented diotically through MDR-900 headphones (Sony Co. Ltd., Tokyo, Japan).

### Stimuli

**Figure 2.**
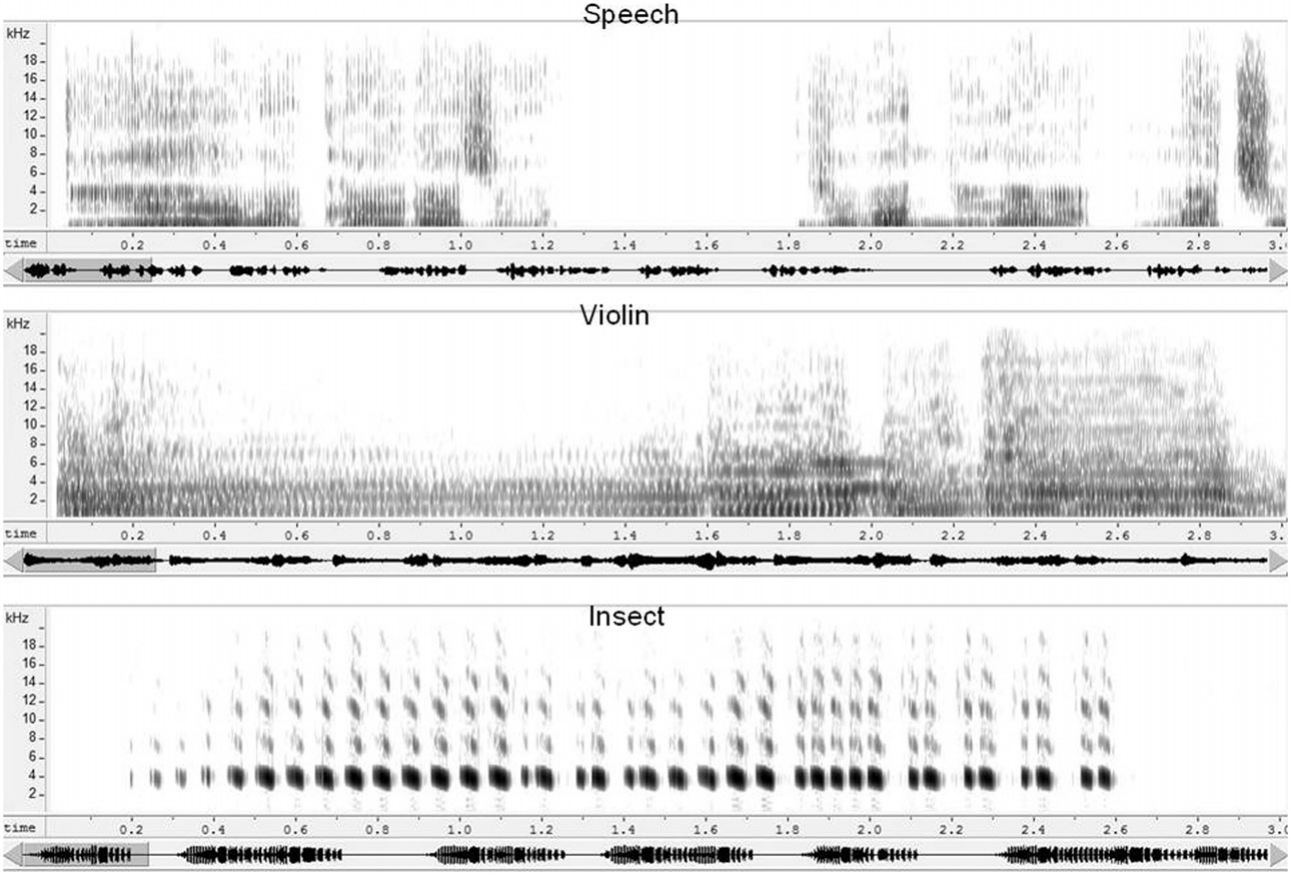
Sound spectrograms of the three test stimuli. Spectograms represent, from top to bottom, the JL, WM, and IS stimuli. The trace below each spectrogram represents the time waveform for each 27.5-s stimulus. The spectrograms each represent the first 3 s of the stimulus, indicated by the shaded area on the waveform trace.

Four different auditory stimuli, each of 29.7-s duration, were presented to each participant. The stimulus set comprised: (1), a spoken passage in Japanese-language (JL), (2), a segment of Western violin music (WM), (3), a recording of chirping insect (cricket) sound (IS), and (4) a passage of sound from a Koto (a Japanese stringed instrument). The Koto sound was used as a reference stimulus. In preliminary studies, a silence was used to obtain a reference measurement, but some of the volunteers fell asleep during the recordings, so the Koto sound was used instead. All were recorded at 44 100 samples/s. Stimulus levels, calculated with silent segments (less than −44 dB) excluded, were equalized in rms (root mean square) level. All stimuli were presented at the same comfortable level. Figure 2 shows spectrograms for the JL, WM, and IS stimuli.

### Procedures

To satisfy circasemidian conditions, all participants were tested on a weekday afternoon after 14:00 at the National Hospital Organization, National Institute of Sensory Organs, Tokyo Medical Center. Each subject was tested in a single session lasting ∼15 min, during which they listened to the acoustic stimuli while sitting inside a soundproof chamber (AT-81, Rion, Tokyo, Japan). The automatic slideshow function was used with sound output reproduction. The time between successive stimulus onsets was 30 s. The variation in the accuracy of presentation duration was less than 0.2 s. The four stimuli were presented, once each, in the following order: JL, WM, IS, Koto. This sequence was repeated 6-times. Data were collected for 30 s per stimulus, resulting in 720 s per volunteer.

We instructed all volunteers to obey the following instructions: (1) Take a seat and listen to the auditory stimuli, (2) Remain still, placing your hands on the table and feet on the floor, and (3) Avoid crossing your arms or feet during the experiment. These instructions were based on previous reports indicating that the subject’s posture could affect the outcome of this study [8,9]. To check that these instructions were followed, each session was monitored via a window from outside the chamber, and via a video camera. Furthermore, the angular velocity sensor was used to detect slight head inclinations and/or other motion. Data from subjects who were found not to be able to follow the instructions were removed from the analysis.

At the end of each session, subjects were asked whether they could identify the audio stimuli. Data from subjects who failed to do so were removed from the analysis.

In our final step we removed data from subjects who displayed the same brain dominance (left or right) for both JL and WM. Among the 70 volunteers, 14 of them fell asleep or otherwise failed to follow instructions or could not identify the three test stimuli (JL, WS, IS). Furthermore, dominance of the cerebral blood flow was not identified in 23 volunteers. Thus, in all, 37 of the original 70 volunteers were excluded.

### Analysis

The goal of this study was to compare the NIRS signal levels recorded during the JL WM, and IS stimuli, with the Koto sound acting as a reference stimulus. For each of the four stimulus types, six 2-channel data blocks of 30 s each were extracted after the trigger points. Data from the second to sixth trials were averaged. The means of the last 25 s were calculated for dual-channel data separately in each averaged block. For each of the three test conditions, the residual of the 25-s mean from the 25-s mean of the reference stimulus was adopted as the stimulus-related activation level.

A laterality index has been suggested in the literature to quantify hemispheric difference in activation level [14]. Based on this, we define a dominance index, DI, for this study:

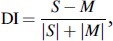

where S/M is the activation level of speech-/music-dominant side; and DI is defined to be positive if the speech hemisphere was dominant and negative if the music hemisphere was dominant.

## Results

**Figure 3.**
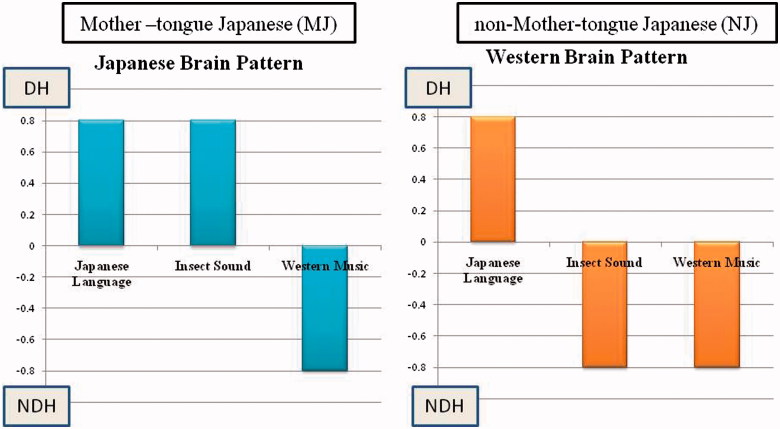
Typical pattern of hemispheric dominance index (DI) for three stimuli. Positive value means dominance and negative value means non-dominance.

Results of subjects who showed dominance indices of opposite sign for the JL and WM stimuli specifically were documented. The side that displayed increased blood flow triggered by the JL stimulus was considered to be the dominant hemisphere (DH). The side that showed an increase prompted by the WM stimulus was considered to be the non-dominant hemisphere (NDH). The purpose of this study was to identify the laterality of cerebral blood flow when the subjects heard the IS stimulus. In the Japanese brain pattern cerebral blood flow increases in the DH when IS was heard while in the Western (non-Japanese) pattern the cerebral blood flow increases in the NDH when IS was heard. Figure 3 is a schematic illustration of the expected patterns of DIs for the three stimuli that would be expected in the two types of dominance pattern (Japanese and Western). Both types show positive DI for Japanese language and negative DI for Western Music. For Insect sound, DI is positive (as for JL) in the Japanese pattern and DI is negative (as for WM) in the Western pattern.

Among the 20 native Japanese speakers (MJ), 16 (80%) exhibited the Japanese brain pattern (JL and IS located in DH), and four (20%) exhibited the Western brain pattern (WM and IS located in NDH). On the other hand, we found that five (38%) of 13 non-native Japanese speakers (non-MJ Caucasian) showed the Japanese pattern, and eight (62%) showed the Western pattern (Table 1). Using Fisher’s exact test, significant differences were observed (*p* = 0.018).

**Table 1.** Contingency table of hemispheric dominance pattern between groups.

	Dominance pattern		
Group	Japanese	Western	Sum
Japanese MJ (Mother Tongue Japanese)	16	4	20
Caucasian NJ (Mother Tongue non-Japanese)	5	8	13
Total	21	12	33

**Figure 4.**
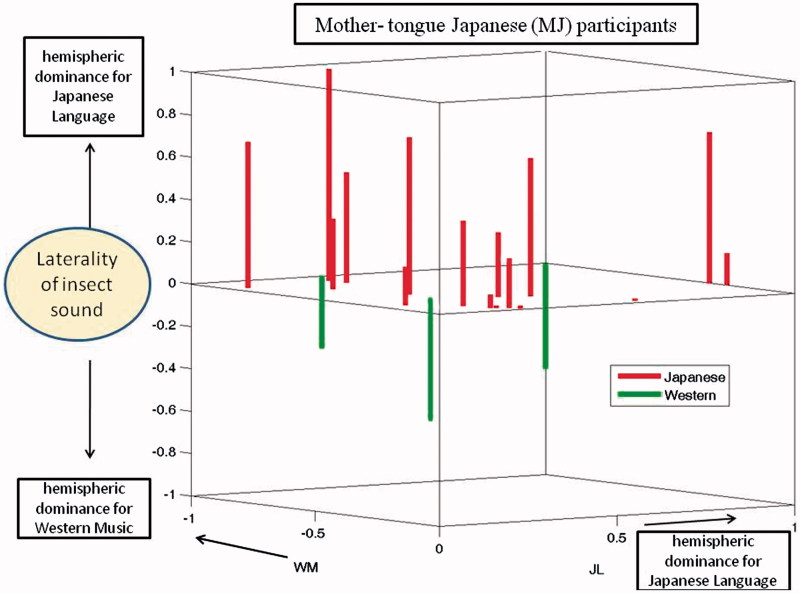
Hemispheric dominance indices (DIs) for three stimuli (Mother-tongue Japanese). The three axes represent the dominance indices for the JL, WM, and IS stimuli. Each bar represents data from one MJ participant. The origin of each bar on the central plane indicates the DI values for JL and WM stimuli. The length of the bar indicates the DI value of the IS stimulus. Participants exhibiting the Japanese pattern are represented by red upward pointing bars, while those exhibiting the Western pattern are represented by green downward pointing bars. There were four participants exhibiting the Western pattern, but only three bars are visible. This is because two subjects both had DIs of 1 and −1 for JL and WM stimuli, respectively.

**Figure 5.**
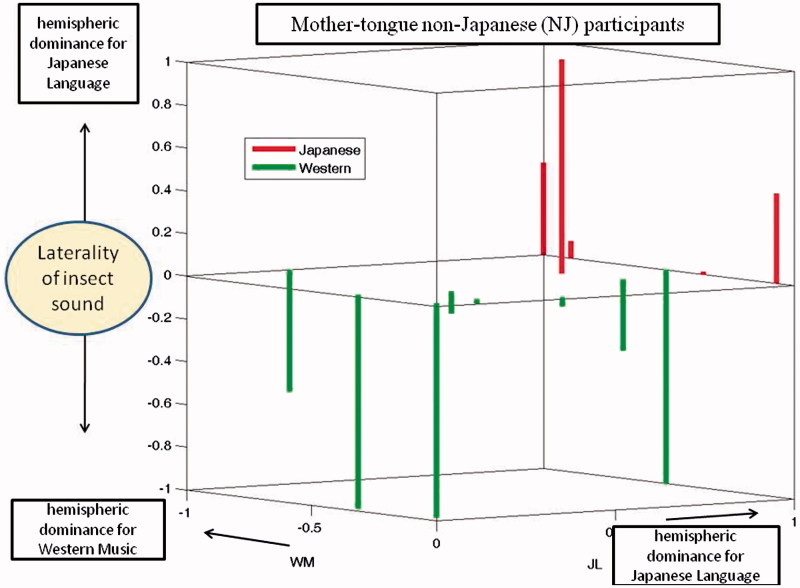
Hemispheric dominance indices (DIs) for three stimuli (non-Mother-tongue Japanese). As for Figure 5, but representing the non-MJ participants.

Dominance indices for the MJ and non-MJ participants are shown in Figures 4 and 5, respectively. These are 3-dimensional plots with indices for the JL and WM stimuli indicated by the axes of the horizontal plane. Results for the IS stimuli are plotted as bars, red pointing up for the Japanese pattern and green pointing down for the Western pattern. It is evident that each of these plots includes some small DIs, indicated by bars that are either very short or originating near the origins of the horizontal axes. To further evaluate the significance of results, we excluded participants whose dominance patterns contained DIs that were not very prominent. When the threshold for prominence (for any of the three test stimuli) was set to 0.07, the remaining group was composed of 10 Japanese-pattern MJs, four Western-pattern MJs, four Japanese-pattern non-MJs, and four Western-pattern non-MJs. Thus, for the MJs, 71% exhibited the Japanese pattern and 39% exhibited the Western pattern, both equally distributed among males and females. For the non-MJs, 50% exhibited the Japanese pattern and 50% exhibited the Western pattern. When the threshold for acceptance was increased from 0.07 to 0.2, the number of remaining participants was 12 MJs (nine Japanese-pattern and three Western-pattern) and five non-MJs (two Japanese pattern and three Western patterns). Thus, the tendency for MJ participants to exhibit the Japanese pattern remained consistent, but the tendency for non-MJ participants to consistently use the Western pattern was not confirmed.

## Discussion

According to Tsunoda [3,5,6] the major difference between the Japanese and Western brain patterns lies in laterality in hemispheric dominance for vowels. The Japanese brain processes isolated vowels as verbal sounds in the verbal hemisphere, but the Western brain processes them as non-verbal sounds in the non-verbal hemisphere. According to Tsunoda’s key-tapping method, native Japanese speakers handled syllables and insect sounds in the verbal brain (DH: Dominant hemisphere), and Western music in the contralateral brain (NDH: Non-dominant hemisphere). Nonnative Japanese speakers handled syllable sounds in the DH, and Western music and insect sounds in the NDH.

While Tsunoda’s key-tapping method used extremely short stimuli (0.1–0.3 s), we compared the differences in brain activity when subjects heard the different long auditory stimuli through both ears with consistent volume and quality. A spoken passage of Japanese was recorded for the JL stimulus, a section of classical music played by the violin for the WM stimulus, and a recording of insects chirping for the IS stimulus (each 29.7-s long).

When informal observations were made during a previous experiment using an fMRI system [15,16], many participants were unable to distinguish IS from the background noise produced by the fMRI. In the present study, however, it was possible to conclude that the majority of native Japanese speakers respond to the IS (insect) auditory stimuli in the language brain. There was a less significant tendency for non-native Japanese speakers (native Western language speakers) to respond to IS in the music brain.

Tsunoda’s theory states that brain dominance in the processing of insect sounds depends on one’s native language and its associated culture. From this conclusion he developed a theory of mental structure and vowels, stating that the mother tongue one acquires in childhood is closely linked with the formation of the unique culture and mentality of each ethnic group [3,5–9]. Interestingly, among the four MJ participants who displayed the Western brain pattern, two of them had become English speakers at an early age. They had been in the US or UK when they were 3–6 years old and attended school with native citizens.

We deem it important that we perform this study with other native Asian or African speakers. We attempted to do so with 11 other Asian volunteers. A massive amount of information has accumulated in the past decade regarding the relationship between language and brain laterality [17]. These findings depend mostly on pre-conscious and automatic processing of short or transitive verbal stimuli. Recent studies have revealed rather bilateral activities in covert higher-order language processing through the sensory-motor co-ordination [18]. The shift in lateralization may be explained by another aspect of brain maturation and short-term skill acquisition that leads to covert left-lateralized language processing [19]. The results just compare the changes in frontal lobe oxy-hemoglobin levels, instead of cerebral blood flow. Further study is necessary to examine the NIRS of the entire brain including Broca’s area. However, this report suggests that we are able to detect the dominant side in language processing for clinical use.

## Conclusions

**Figure 6.**
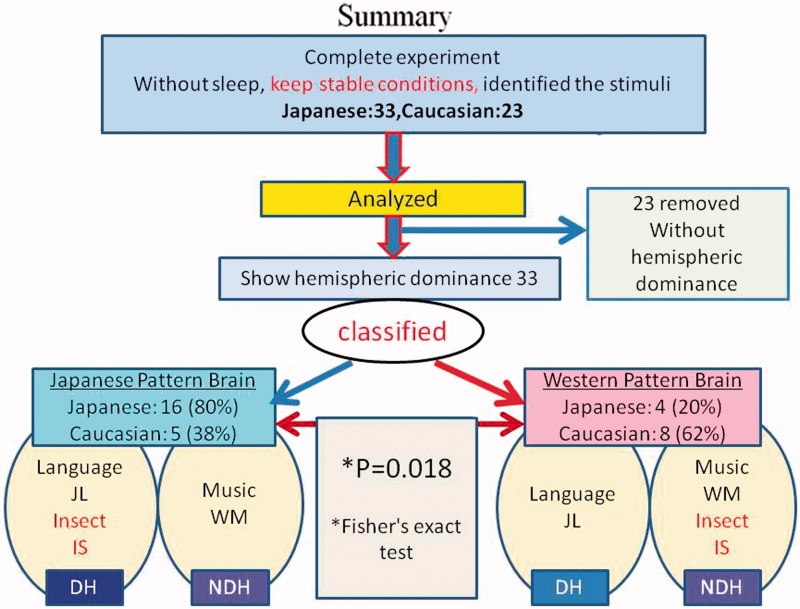
Schematic summarizing the results (before application of a DI threshold) and the participant-selection procedures leading up to them.

Using NIRS, we compared the differences in both cerebral hemispheres in response to three auditory stimuli in native and non-native speakers of Japanese in a more natural experimental setting than previous studies. We concentrated only on subjects who showed clear laterality differences for spoken and musical sounds. The results confirmed that, among these subjects, native Japanese speakers tended (80%) to process IS in the same cerebral hemisphere as they do JL. Non-native Japanese speakers tended (62%) to process IS in the opposite hemisphere from that which processes JL. Fisher’s exact test, using these results, confirmed that these tendencies are significant. The steps leading up to this result are summarized in Figure 6.

When a stricter criterion for laterality was imposed, results for the remaining native Japanese speakers remained strong (71% showed the Japanese pattern), while the results for the non-native Japanese speakers did not exhibit any tendency. These results partially support those obtained previously using Tsunoda’s methods [1,3–7] concerning dominancy in the perception of insect sounds, although further data would have to be obtained for the male and female non-native Japanese speakers.
